# Blood Plasma Small Non-Coding RNAs as Diagnostic Molecules for the Progesterone-Receptor-Negative Phenotype of Serous Ovarian Tumors

**DOI:** 10.3390/ijms241512214

**Published:** 2023-07-30

**Authors:** Angelika V. Timofeeva, Ivan S. Fedorov, Aleksandra V. Asaturova, Maya V. Sannikova, Anna V. Tregubova, Oleg A. Mayboroda, Grigory N. Khabas, Vladimir E. Frankevich, Gennady T. Sukhikh

**Affiliations:** 1 National Medical Research Center for Obstetrics, Gynecology and Perinatology Named after Academician V.I. Kulakov Ministry of Healthcare of the Russian Federation, Ac. Oparina 4, 117997 Moscow, Russia; is_fedorov@oparina4.ru (I.S.F.); a_asaturova@oparina4.ru (A.V.A.); m_sannikova@oparina4.ru (M.V.S.); a_tregubova@oparina4.ru (A.V.T.); g_khabas@oparina4.ru (G.N.K.); v_frankevich@oparina4.ru (V.E.F.); g_sukhikh@oparina4.ru (G.T.S.); 2 Center for Proteomics and Metabolomics, Leiden University Medical Center, Postbus 9600, 2300 RC Leiden, The Netherlands; oleg00m2work@gmail.com; 3 Laboratory of Translational Medicine, Siberian State Medical University, 634050 Tomsk, Russia; 4 Department of Obstetrics, Gynecology, Perinatology and Reproductology, First Moscow State Medical University Named after I.M. Sechenov, 119991 Moscow, Russia

**Keywords:** miRNA, piRNA, mRNA, CA125, progesterone receptor (PGR), new-generation sequencing (NGS), quantitative RT-PCR, serous ovarian carcinoma, borderline cystadenoma, benign cystadenoma, formalin-fixed paraffin-embedded (FFPE) blocks, blood plasma, cytoreduction

## Abstract

The expression level of the progesterone receptor (PGR) plays a crucial role in determining the biological characteristics of serous ovarian carcinoma. Low PGR expression is associated with chemoresistance and a poorer outcome. In this study, our objective was to explore the relationship between tumor progesterone receptor levels and RNA profiles (miRNAs, piwiRNAs, and mRNAs) to understand their biological characteristics and behavior. To achieve this, we employed next-generation sequencing of small non-coding RNAs, quantitative RT-PCR, and immunohistochemistry to analyze both FFPE and frozen tumor samples, as well as blood plasma from patients with benign cystadenoma (BSC), serous borderline tumor (SBT), low-grade serous ovarian carcinoma (LGSOC), and high-grade serous ovarian carcinoma (HGSOC). Our findings revealed significant upregulation of *MMP7* and *MUC16*, along with downregulation of *PGR*, in LGSOC and HGSOC compared to BSC. We observed significant correlations of PGR expression levels in tumor tissue with the contents of miR-199a-5p, miR-214-3p, miR-424-3p, miR-424-5p, and miR-125b-5p, which potentially target *MUC16, MMP7*, and *MMP9*, as well as with the tissue content of miR-16-5p, miR-17-5p, miR-20a-5p, and miR-93-5p, which are associated with the epithelial–mesenchymal transition (EMT) of cells. The levels of EMT-associated miRNAs were significantly correlated with the content of hsa_piR_022437, hsa_piR_009295, hsa_piR_020813, hsa_piR_004307, and hsa_piR_019914 in tumor tissues. We developed two optimal logistic regression models using the quantitation of hsa_piR_020813, miR-16-5p, and hsa_piR_022437 or hsa_piR_004307, hsa_piR_019914, and miR-93-5p in the tumor tissue, which exhibited a significant ability to diagnose the PGR-negative tumor phenotype with 93% sensitivity. Of particular interest, the blood plasma levels of miR-16-5p and hsa_piR_022437 could be used to diagnose the PGR-negative tumor phenotype with 86% sensitivity even before surgery and chemotherapy. This knowledge can help in choosing the most effective treatment strategy for this aggressive type of ovarian cancer, such as neoadjuvant chemotherapy followed by cytoreduction in combination with hyperthermic intraperitoneal chemotherapy and targeted therapy, thus enhancing the treatment’s effectiveness and the patient’s longevity.

## 1. Introduction

According to global statistics, ovarian cancer is ranked seventh in terms of cancer mortality among women [1]. While the mortality rate of ovarian cancer has decreased by over 30% in the past 50 years thanks to advancements in treatment, the survival rate remains below 50% at 5 years after diagnosis [2]. The primary treatment approaches for advanced ovarian cancer involve a combination of surgery and chemotherapy (combining paclitaxel and platinum drugs with bevacizumab or PARP inhibitors), though the optimal order of these treatments is yet to be determined.

Cancer antigen 125 (CA125) is commonly used as a marker for ovarian cancer. However, elevated levels are detected in only 50% of disease stage I cases and 80% of disease stage III-IV cases. CA125 levels should be interpreted alongside clinical signs and ultrasound findings due to nonspecificity and occurrence in other diseases [3,4,5,6]. CA125 is utilized to monitor a patient’s response to neo-adjuvant and adjuvant treatments [7,8], or to predict the overall survival probability at 3 months after the completion of primary treatment. If a CA125 value is above 35, the risk of death is 51% at 24 months and can go up to 79% at 60 months [9].

Improvements in the early-stage diagnosis of invasive serous ovarian/tubal carcinoma have resulted from two fully completed, large-scale population clinical trials using a multimodal screening strategy (measuring serum CA125 levels and conducting transvaginal ultrasounds) [10,11]. However, despite these efforts, there has been no significant reduction in ovarian cancer mortality compared to the no-screening cohort.

Various research teams are currently developing new strategies for ovarian cancer screening, focusing on the selection of biomarkers alone or in combination with CA 125, such as ROMA, CPH1, OVA1, and Overa [12,13]. These approaches aim to improve the detection of early-stage disease and reduce ovarian cancer mortality. However, the aggressive behavior of a tumor is influenced by its biological properties, such as an extensive stromal reaction and increased invasiveness, which contribute to the failure of cytoreductive surgery and chemo-resistance [14].

Recent studies have revealed that the hormone receptor status plays a crucial role in defining tumor invasiveness and longevity. Specifically, low levels of progesterone receptor (*PGR*) expression are associated with a more aggressive disease course and worse outcomes in LGSOC and HGSOC [15,16], as well as in endometrioid carcinoma [17]. On the contrary, patients with poorly differentiated epithelial ovarian tumors show an improved survival rate when associated with high serum progesterone levels along with the expression of *PGR* [18]. This protective effect of progesterone may be partly due to the PGR-mediated suppression of progesterone receptor membrane component-1, which enhances the sensitivity of ovarian cancer cells to platinum-based chemotherapy [19].

The main regulators of signaling pathways within a cell, controlling gene expression at the transcriptional and/or post-transcriptional levels, are small non-coding RNAs, including microRNAs (miRNAs) and piwiRNAs (piRNAs). These two types of RNAs differ in their genomic location, biogenesis, length of functionally active molecules, and mechanisms of action [20,21,22]. Specifically, they bind to two different subfamilies of Argonaute proteins (AGO-clade for miRNAs and PIWI-clade for piRNAs) to guide target-specific gene regulation. The precursor molecule of piRNAs is single-stranded, in contrast to the double-stranded hairpin structure of miRNA precursors. Additionally, the biogenesis of piRNAs is Dicer-independent, and miRNAs are slightly smaller (18–25 nt) than piRNAs (25–32 nt). Moreover, piRNAs display much greater sequence diversity (for humans, there are 8,438,265 piRNA sequences according to the piRBase v.2.0 database compared to 2600 miRNA sequences according to miRBase v.22). A unique feature of piRNAs is their regulation of cell genome stability by suppressing the activity of mobile genetic elements, such as transposons. Furthermore, piRNAs can control gene expression not only at the post-transcriptional level, as miRNAs do, but also at the transcriptional level through DNA methylation and histone modifications.

Numerous miRNAs have been identified [23,24] as potential contributors to the pathogenesis of gynecological diseases, displaying distinct histotype-specific patterns [25]. Additionally, the role of piRNAs in various types of cancers has been demonstrated [26]. Recently, it has been shown that PIWI proteins and piRNAs play a prometastatic role in ovarian carcinoma, contributing to disease progression, and are being considered as potential diagnostic and prognostic biomarkers for ovarian cancer [27,28]. MiRNAs and piRNAs have also been identified in peripheral blood as markers associated with clinicopathological features of different cancer types [20]. However, despite the progress in this field, their clinical application requires further validation using independent large test samples.

The present study aimed to explore the relationship between the level of progesterone receptor expression in serous tumors and small non-coding RNAs, which may act as potential regulators of CA125 and epithelial–mesenchymal transition, influencing the development of an aggressive tumor phenotype and chemoresistance. Developing a liquid biopsy method to diagnose progesterone-receptor-negative serous ovarian cancer through the quantitative evaluation of miRNAs and piRNAs in peripheral blood, rather than in cancer tissue, is of great interest for the appropriate management of patients with this type of primary tumor before initiating any treatment.

## 2. Results

### 2.1. Analysis of Tumor-Specific miRNAs That Regulate the Level of CA125 in Blood Serum

To identify potential mechanisms of changes in CA125 secretion levels in patients with serous ovarian tumors, we conducted a comparison between miRNA sequencing data from tumor tissue (Table S1) and miRWalk data (http://mirwalk.umm.uni-heidelberg.de/ (accessed on 1 February 2023)). The analysis involved a comparison of miRNA expression patterns in HGSOC tissue samples from patients P25, P26, and P28 (Table 1) with benign cystadenoma tissue samples from patients P4, P6, and P7 (Table 1), leading to the identification of 144 differentially expressed miRNAs (Table S1, Sheet 1). Among these miRNAs, 64 were significantly downregulated (Table S1, Sheet 2). According to miRWalk, 31 out of these 64 miRNAs (Table S1, Sheet 3) are potential regulators of the expression levels of mucin 16 (*MUC16*), matrix metalloproteinase 7 (*MMP7*), and matrix proteinase 9 (*MMP9*).

It is important to note that MMP7 and MMP9 are believed to cleave the extracellular domain of MUC16, resulting in the release of CA125 [29]. The increased expression and activity of MMP7 and MMP9 create conditions favorable for the metastasis of ovarian cancer cells by remodeling the extracellular matrix, enhancing their migration, and facilitating attachment to secondary sites [30]. Specifically, upregulation of *MMP7* can be triggered by the interaction between the carboxy-terminal portion of the MUC16/CA125 protein expressed in ovarian cancer cells and mesothelin present on mesothelial cells, leading to an increase in invasive tumor properties and peritoneal carcinomatosis [31,32,33].

We further analyzed the levels of the following miRNAs in FFPE sections of tumor tissue from 38 patients (Table 1) using real-time quantitative PCR (Table S5): hsa-miR-199a-5p, hsa-miR-424-3p, hsa-miR-424-5p, hsa-miR-134-5p, hsa-miR-214-3p, hsa-miR-125b-5p, and hsa-miR-139-5p.

**Figure 1:**
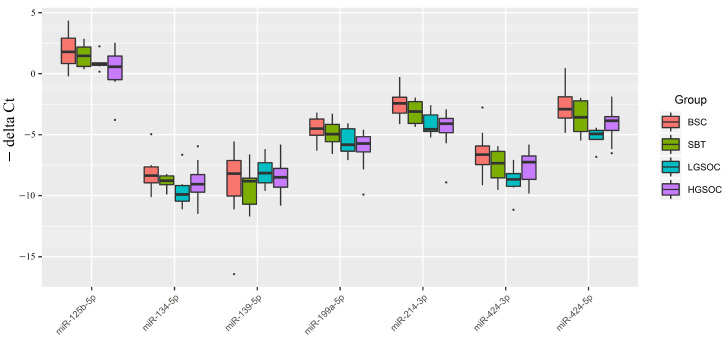
Boxplot of the expression level of miRNAs potentially regulating the level of CA-125 in serous ovarian tumors.

The relative miRNA expression level was calculated from the difference between the threshold cDNA amplification cycles (Ct) of the analyzed miRNA and the reference endogenous SNORD68 (Figure 1). Analysis of the significance of differences in miRNA expression level (−∆Ct) in the compared groups was performed using a two-sided Wilcoxon–Mann–Whitney test (Table 2).

From Figure 1 and Table 1, it is evident that the median expression levels of the analyzed miRNAs in malignant ovarian neoplasms (SBT, LGSOC, and HGSOC) were lower than those in benign serous ovarian tumors (BSC), which aligns with the sequencing data presented in Table S1. Notably, a significant downregulation of miR-214-3p and miR-424-5p, which potentially regulate *MMP7* and/or *MUC16*, was observed in the groups of serous ovarian carcinomas (LGSOC and HGSOC). Additionally, in the HGSOC group, miR-125b-5p and miR-199a-5p were identified as potential additional regulators of the expression levels of *MUC16* and *MMP7*, as their expression levels were significantly reduced compared to the BSC group.

It is important to highlight that only the LGSOC group, when compared to the BSC group, displayed a significant decrease in the expression levels of miR-134-5p and miR-424-3p, which are potential regulators of the expression levels of target genes *MUC16* and *MMP9*. However, no significant differences in the expression levels of all analyzed miRNAs were observed between the SBT and BSC groups.

### 2.2. Analysis of MMP7, MMP9, MUC16, and PGR Gene Expression Levels in Serous Ovarian Tumors

**Figure 2:**
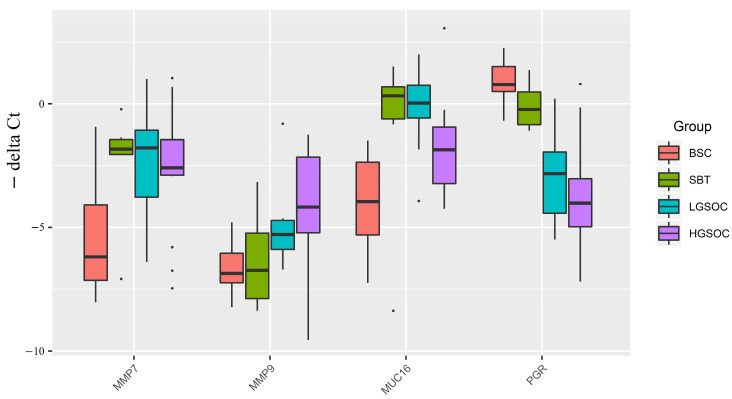
Boxplot of *MMP7*, *MMP9*, *MUC16*, and *PGR* mRNA expression levels in serous ovarian tumors.

The expression levels of the *MMP7*, *MMP9*, *MUC16*, and *PGR* genes were analyzed in 38 samples of FFPE sections of serous tumors, including BSC, SBT, LGSOC, and HGSOC (Table 1), using quantitative real-time PCR with *GAPDH, TUBA1B*, and *ACTNB* as reference genes (Figure 2, Table 3 and Table S5). Among the malignant serous tumors (SBT, LGSOC, and HGSOC), there was a trend toward increased expression of *MMP7*, *MMP9*, and *MUC16* mRNAs, and a trend toward decreased expression of PGR mRNA, compared to benign serous tumors (BSC). Furthermore, a significant increase in the expression levels of *MMP7*, *MMP9*, and *MUC16* genes was observed in the serous ovarian carcinoma groups (LGSOC and HGSOC) when compared to the BSC group (Table 3). Additionally, the SBT group showed a significant increase in the expression levels of *MMP7* and *MUC16* compared to the BSC group (Table 3).

In the HGSOC group, there were opposite significant changes in the expression levels of *MMP7* and *MUC16* genes (upregulation) and tissue content of their potential regulators miR-214-3p, miR-424-5p, miR-125b-5p, and miR-199a-5p (downregulation) (Figure 1 and Figure 2). Similarly, in the LGSOC group, there was significant upregulation of the *MMP7, MMP9*, and *MUC16* genes, and downregulation of their potential regulators (miR-214-3p, miR-424-5p, miR-134-5p, and miR-424-3p) (Figure 1 and Figure 2).

Importantly, alongside the significant increase in the expression levels of *MMP7, MMP9*, and *MUC16*, there was a significant decrease in the expression level of *PGR* in the LGSOC and HGSOC samples (Figure 2). This observation suggests that the reduced expression of *PGR* may contribute to the invasive properties of the tumor and its ability to metastasize.

### 2.3. Analysis of microRNA Regulators of EMT in Serous Ovarian Tumors Tissue

**Figure 3:**
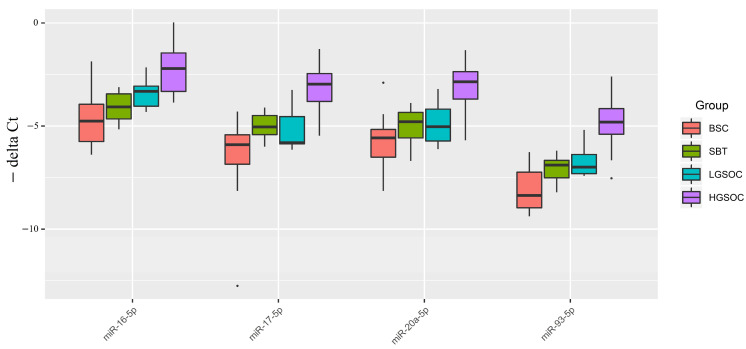
Boxplot of the expression level of miRNAs potentially regulating EMT in serous ovarian tumors.

In a previous study [16], we detected differential expression of EMT-associated miR-16-5p, miR-17-5p, miR-20a-5p, and miR-93-5p in the blood plasma of PGR-negative HGSOC patients. The present study aimed to analyze the expression level of these miRNAs in FFPE sections of serous tumors as a function of PGR expression level. The relative level of miRNA expression was calculated from the difference between the threshold cDNA amplification cycles of the analyzed miRNA and the reference endogenous SNORD68 (Figure 3).

Figure 3 and Table 4 indicate that, in malignant ovarian tumors (SBT, LGSOC, and HGSOC), the median expression levels of miRNAs potentially regulating EMT were higher than those in benign serous ovarian tumors (BSC). This finding is consistent with the miRNA sequencing data in serous tumor tissues (Table S1, Sheet 1, and Sheet 4). Specifically, a significant increase in the expression levels of miR-16-5p, miR-17-5p, miR-20a-5p, and miR-93-5p was observed in HGSOC relative to BSC. In the LGSOC group, only miR-16-5p showed a significant upregulation. However, no significant changes in the analyzed miRNAs responsible for EMT were observed in the SBT group.

The upregulation of miRNAs responsible for EMT, along with the decreased expression level of PGR and increased expression levels of MMP7 and MUC16, coupled with opposite changes in their potential regulators (miR-214-3p, miR-424-5p, miR-125b-5p, and miR-199a-5p), may suggest a more aggressive behavior of HGSOC concerning its ability to metastasize in comparison to SBT and LGSOC.

### 2.4. Analysis of piRNA Expression in Serous Ovarian Tumors

One of the essential functions of piRNAs is to regulate the stability of the cell genome. They achieve this by interacting with retrotransposon transcripts in the nucleus as part of the RISC complex. This complex then associates with histone deacetylase, histone methyltransferase, and DNA methyltransferase to inhibit the transcription of retrotransposons. This process effectively prevents their activity and integration into various regions of the genome [22].

Beyond their suppressive activity against transposons, piRNAs also exert regulatory effects on various signaling pathways within the cell. This can occur through the destabilization or inhibition of target mRNA translation, as well as the stabilization or activation of target mRNA translation [22,34]. Considering the proven genomic instability and alterations in the activity of numerous signaling pathways observed in cancer cells, particularly in serous ovarian carcinomas [15,35,36,37,38,39], we conducted an analysis of piRNA expression profiles in SBT tissue from patients P13, P15, and P16 (Table 1), as well as in HGSOC tissue from patients P25, P26, and P28 (Table 1), in comparison to BSC tissue from patients P4, P6, and P7 (Table 1) using the deep sequencing method (Table S2, Sheet 1, and Sheet 2).

**Figure 4:**
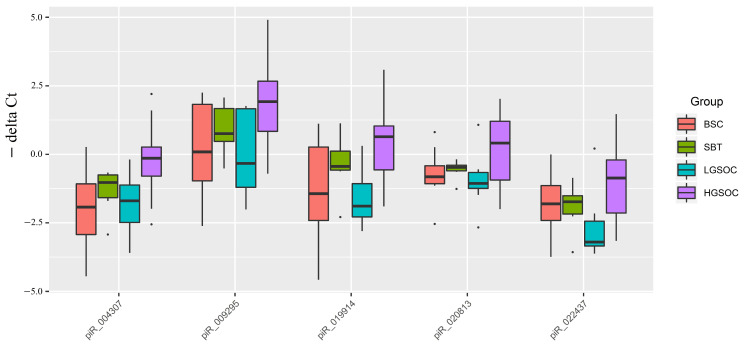
Box plot of piRNA expression levels in FFPE sections of serous ovarian tumors.

Through this analysis, we identified 97 piRNAs and 77 piRNAs that were differentially expressed in the HGSOC and SBT groups, respectively, compared to BSC (*p* < 0.1), with an overlapping list of 37 piRNAs. Further analysis of 38 samples of FFPE tissue sections of serous ovarian tumors led to the selection of 19 piRNAs for quantitative real-time PCR (Table S5). Among these, 6 piRNAs showed altered expression levels in both HGSOC and SBT, while the remaining 13 piRNAs displayed altered expression levels solely in HGSOC. The relative expression levels of piRNAs were calculated based on the difference between the threshold cycles of cDNA amplification of the analyzed piRNA and the reference endogenous SNORD68 (Figure 4).

Out of 19 piRNAs, a Ct value of less than 35 cycles was observed only in hsa_piR_004307, hsa_piR_009295, hsa_piR_019914, hsa_piR_020813, and hsa_piR_022437, among which significant differences were found for hsa_piR_004307, hsa_piR_009295, and hsa_piR_019914 in the HGSOC group relative to BSC (Table 5).

### 2.5. Correlation Analysis of the Expression Level of Tumor-Associated miRNA, piRNA, mRNA, Progesterone Receptor, and the Level of CA125 in the Blood Serum of Patients

**Figure 5:**
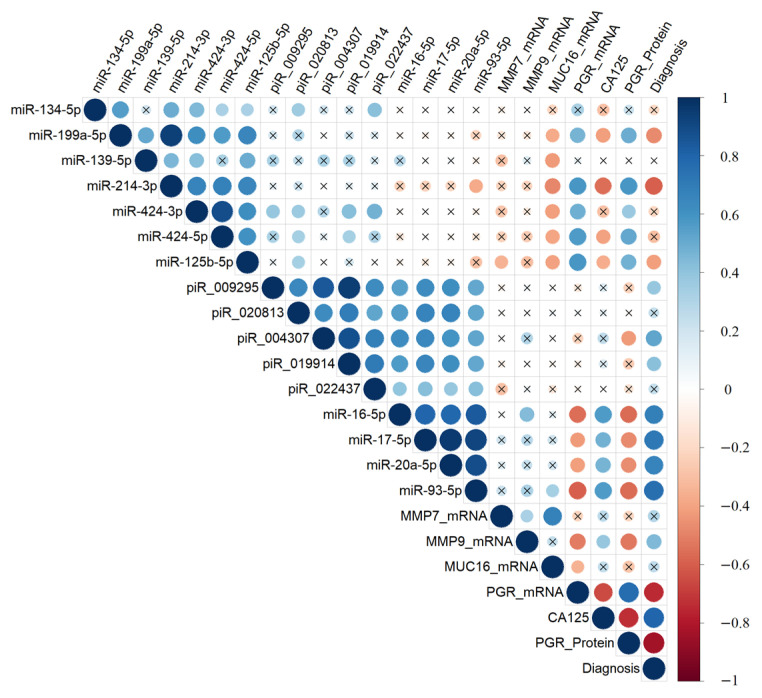
Correlation analysis of the miRNA, piRNA, and mRNA expression levels, as well as PGR protein level, in 38 FFPE samples of serous tumors, and corresponding patient’s blood serum CA125 level. Dot means significant correlations (*p* < 0.05), cross means non-significant correlations, direct correlations are highlighted in blue, and inverse correlations are highlighted in red. The larger the size of the dot, the more significant the correlation. The analyzed samples were arranged according to the diagnosis (type of the serous tumor) in the following way: “BSC” < “SBT” < “LGSOC” < “HGSOC”.

A Spearman correlation matrix was constructed to explore potential relationships between the characteristics of the ovarian tumor process, forming the molecular-biological portrait of different types of serous tumors (Figure 5, Table S3). The analyzed samples were arranged according to the diagnosis (type of serous tumor) in the following order: “BSC” < “SBT” < “LGSOC” < “HGSOC”.

The expression levels of hsa-miR-199a-5p, hsa-miR-214-3p, hsa-miR-424-5p, and hsa-miR-125b-5p were significantly and inversely correlated with the expression level of *MUC16* mRNA in the tumor tissue and with the CA125 level in the blood serum. However, they showed a direct correlation with the level of *PGR* mRNA and PGR protein (according to Allred score). On the other hand, the expression levels of miR-16-5p, miR-17-5p, miR-20a-5p, and miR-93-5p were significantly and inversely correlated with the level of PGR. Conversely, they were directly correlated with the expression levels of hsa_piR_004307, hsa_piR_009295, hsa_piR_019914, hsa_piR_020813, and hsa_piR_022437 in the tumor tissue, and with the CA125 level in the blood serum of patients.

Among the piRNAs, the expression level of hsa_piR_004307 was significantly and inversely correlated with the PGR protein level in the tumor tissue. Furthermore, *MMP7* mRNA was significantly and directly correlated with the level of *MUC16* mRNA and *MMP9* mRNA. In contrast, *MMP9* mRNA was inversely correlated with the level of PGR. Notably, the level of *PGR* mRNA showed a significant correlation with the level of PGR protein, both of which were significantly and inversely correlated with the level of CA125 in the blood serum of patients with serous ovarian tumors.

### 2.6. Partial Least Squares Discriminant Analysis (PLS-DA) of the Molecular Biological Parameters Determining Certain Types of Tumors

**Figure 6:**
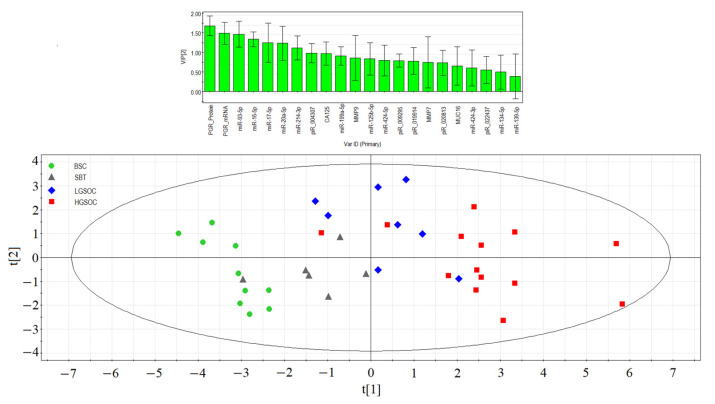
Partial least squares analysis (PLS) of “−ΔCt” RT-PCR data on the expression of miRNA, piRNA, mRNA, and PGR protein in the 38 FFPE samples of serous ovarian tumors and corresponding CA125 level in blood serum of patients. Score plot with the imposition of information of the molecular biological parameter value on the serous tumor type (BSC, SBT, LGSOC, and HGSOC) is presented in the bottom of the figure. Variable Importance in Projection (VIP) score is presented at the top of the figure.

Significant correlations were observed between various molecular biological parameters depending on the type of serous ovarian tumor, prompting an evaluation of each parameter’s contribution to the formation of specific tumor types (BSC, SBT, LGSOC, and HGSOC). All data obtained in Section 2.1, Section 2.2, Section 2.3 and Section 2.4 were utilized for partial least squares (PLS) analysis, and the results are presented in the graph shown in Figure 6. This graph clearly illustrates the formation of distinct clusters of samples depending on the type of serous tumor.

In the separation of BSC, SBT, LGSOC, and HGSOC groups, the molecules with Variable Importance in Projection (VIP) score greater than 1 had the most significant contributions. These key molecules include the expression level of *PGR*, miRNAs responsible for EMT (miR-16-5p, miR-17-5p, miR-20a-5p, and miR-93-5p), hsa_piR_004307 (a potential regulator of genome stability and signaling pathways in the cell), miR-214-3p (a potential regulator of *MMP7* and *MUC16* expression levels), and the CA125 level in patients’ blood serum. Among these, the *PGR* expression level emerged as playing a primary role in the separation of different serous tumor types.

The PLS analysis provided valuable insights into the significant molecular factors that contribute to the distinctive characteristics of BSC, SBT, LGSOC, and HGSOC, helping to better understand the underlying molecular mechanisms behind each tumor type.

### 2.7. Logistic Regression Models for Diagnosing a Progesterone-Receptor-Negative Serous Ovarian Tumor Based on miRNA and piRNA Expression Levels in Tumor Tissue

The relationship between the expression level of *PGR* in tumor tissue and the response to adjuvant chemotherapy was investigated, and the results are presented in Table 1. Specifically, in PGR-negative LGSOC and HGSOC cases, disease stabilization or progression was observed in 75% and 100% of cases, respectively. This indicates that patients with PGR-negative tumors were less responsive to the adjuvant chemotherapy, with a higher likelihood of disease stabilization or progression.

**Figure 7:**
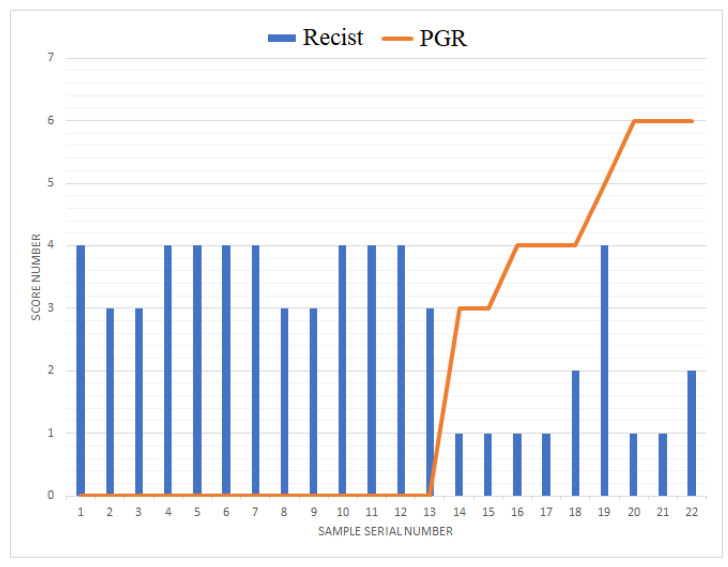
Combined plot of tumor response to chemotherapy according to RECIST 1.1 MRI/CT criteria and progesterone receptor expression level with respect to Allred score in LGSOC and HGSOC.

Figure 7 provides a visual representation of the data, with an overlay of a histogram and a line chart showing RECIST 1.1 MRI/CT criteria data and Allred scores for PGR, respectively. From the figure, it is evident that, in all cases of a PGR-negative tumor, a stable or progressive disease was observed after a course of adjuvant chemotherapy. This reinforces the finding that PGR-negative tumors tend to exhibit a poorer response to adjuvant chemotherapy, potentially indicating a more aggressive disease course in these cases.

Due to the observed correlations between the expression level of *PGR* and the expression levels of small non-coding RNAs (miRNA and piRNA), as well as protein-coding mRNAs that are responsible for the biological properties of specific types of serous ovarian tumors, a logistic regression model was developed for tumor identification based on the PGR-phenotype.

**Figure 8:**
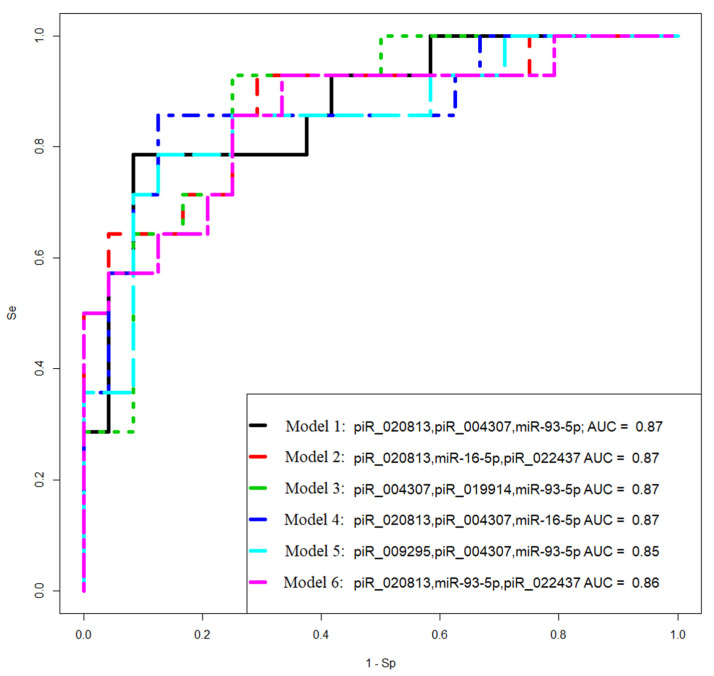
Receiver operating characteristic (ROC) curves of the logistic regression models to identify PGR-negative serous tumors based on the level of miRNA and piRNA in FFPE samples of BSC, SBT, LGSOC, and HGSOC.

To create the logistic regression models, the “−ΔCt” values obtained in Section 2.1, Section 2.2, Section 2.3 and Section 2.4 were used. The models were developed in the RStudio program (Figure 8, Table 6) by determining the optimal combination of predictor variables. The inclusion and exclusion of variables in the model were carried out in a stepwise manner based on their contribution to the model and their statistical significance. In this model, the *PGR* expression level was used as the dependent variable, with the binary classification of tumors into PGR-positive (0) and PGR-negative (1) based on the Allred score.

The logistic regression model aimed to identify the important predictors (miRNA, piRNA, and protein-coding mRNA expression levels) that can help distinguish between PGR-positive and PGR-negative tumors. By analyzing the contribution and significance of these variables, the model can effectively classify serous ovarian tumors based on their PGR phenotype.

Models 2 and 3 in Figure 8 had the highest sensitivity (93%) in identifying PGR-negative tumors.

### 2.8. Logistic Regression Models for the Diagnosis of Progesterone-Receptor-Negative and Chemoresistant Serous Ovarian Tumor by the Level of Tumor-Associated miRNAs and piRNAs Circulating in the Blood of Patients

To diagnose PGR-negative serous ovarian tumors before surgical and chemotherapeutic treatment, the levels of miRNA and piRNA—which form models 2 and 3 in Figure 8—were analyzed using real-time RT-PCR in the blood plasma of 38 patients (identified by their ID numbers in Table 1). The “−ΔCt” values were obtained by using hsa_piR_004308 as a reference molecule to quantify hsa_piR_020813, hsa_piR_022437, hsa_piR_004307, and hsa_piR_019914. Additionally, hsa-miR-30d-5p was used as a reference molecule to analyze the levels of hsa-miR-16-5p and hsa-miR-93-5p.

**Figure 9:**
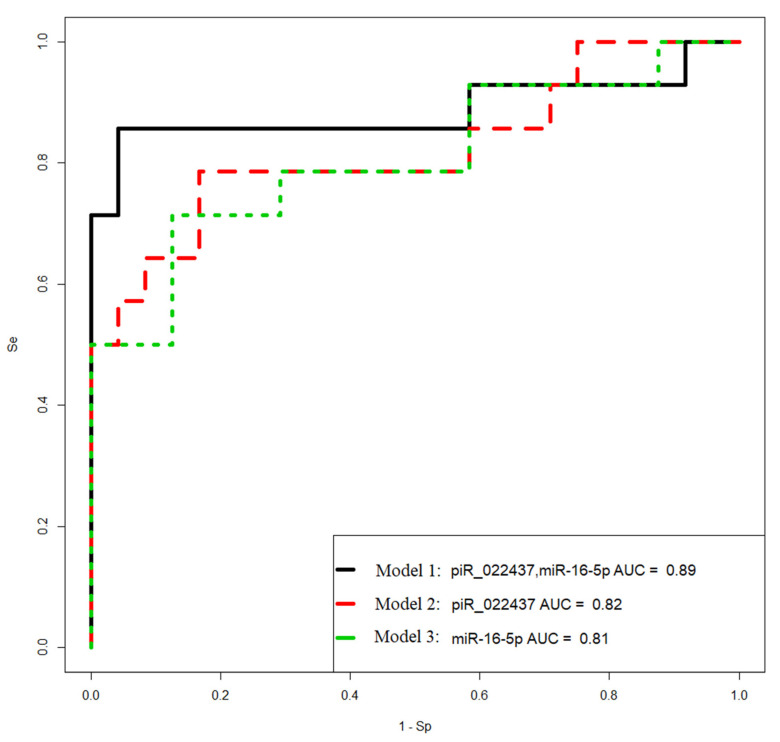
Receiver operating characteristic (ROC) curves of the logistic regression models to identify PGR-negative serous tumors based on the level of miRNA and piRNA in blood plasma samples from patients with BSC, SBT, LGSOC, and HGSOC.

Logistic regression models were developed based on the obtained data and are presented in Figure 9. These models aim to predict the likelihood of PGR-negative serous ovarian tumors based on the expression levels of the selected miRNAs and piRNAs in the blood plasma. The models help identify patients who may have PGR-negative tumors before undergoing surgical and chemotherapeutic treatments, providing valuable information for treatment planning and personalized care.

The parameters of the developed models are presented in Table 7, providing insights into the coefficients and significance of the predictor variables used in the logistic regression analysis. These models contribute to the early detection and diagnosis of PGR-negative serous ovarian tumors, potentially leading to better treatment outcomes and patient management.

Model 1 in Figure 9 has the highest sensitivity (86%) in identifying PGR-negative tumors. The Formula (1) describing this model is presented below:

(1) $$\frac{1}{1+e^{4.3-1.29x_{1}-0.75x_{2}}\;}$$

where *x*_1_ indicates “−ΔCt” for hsa_ piR_022437, and *x*_2_ indicates “−ΔCt” for hsa-miR-16-5p.

Considering the significant finding that PGR-negative serous ovarian tumors are associated with stable or progressive disease after adjuvant chemotherapy, a logistic regression model was developed to predict chemoresistance in LGSOC and HGSOC before any form of treatment. To construct the model, a set of tumor-specific miRNAs and piRNAs from Section 2.1, Section 2.2, Section 2.3 and Section 2.4 were quantified in blood plasma, and the resulting “−ΔCt” values were utilized. These values were then employed in the development of logistic regression models using the RStudio program. In these models, the response of the tumor to chemotherapy was considered the dependent variable, with values of 0 indicating complete or partial response and values of 1 indicating stable or progressive disease according to RECIST 1.1 MRI/CT criteria.

**Figure 10:**
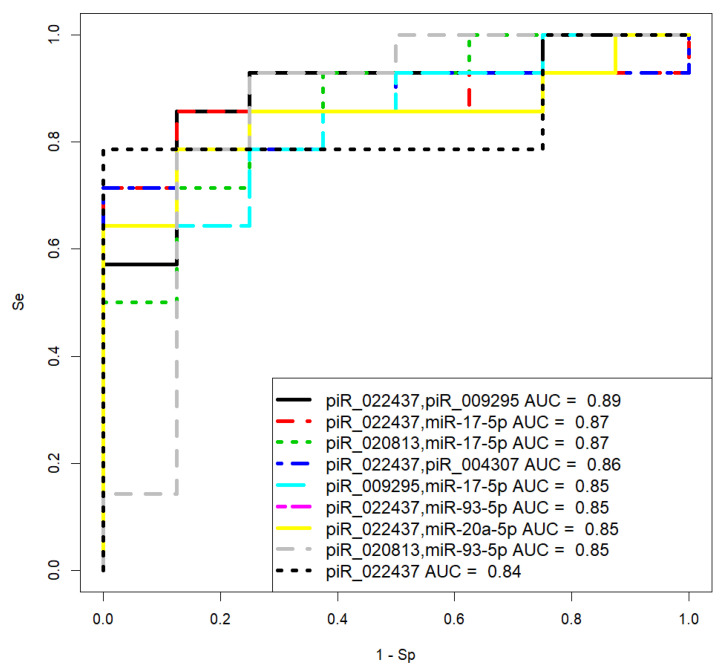
Receiver operating characteristic (ROC) curves of the logistic regression models to prognose chemoresistance of the tumor based on the level of miRNA and piRNA in blood plasma samples from patients with LGSOC and HGSOC.

The logistic regression models and their corresponding parameters are presented in Figure 10 and Table 8, respectively. These models serve as prognostic tools for predicting the likelihood of chemoresistance in LGSOC and HGSOC prior to any treatment intervention. By utilizing the expression levels of the selected miRNAs and piRNAs in the blood plasma, these models provide valuable insights for clinicians to identify patients who may have a higher risk of chemoresistance, thereby enabling the development of more tailored and effective treatment strategies.

Model 3 in Figure 10 has the optimal significance of all predictor variables in comparison to other models and high sensitivity (85.71%) to prognose chemoresistance of the tumor. The Formula (2) describing this model is presented below:

(2) $$\frac{1}{1+e^{-6.29-0.27x_{1}-1.05x_{2}}}$$

where *x*_1_ indicates “−ΔCt” for hsa_ piR_020813, and *x*_2_ indicates “−ΔCt” for hsa-miR-17-5p.

It should be noted that model 9, based on the quantification of piR_022437, has 100% specificity in predicting complete or partial response to chemotherapy, which can be used to decide whether cytoreductive surgery followed by adjuvant chemotherapy is feasible.

### 2.9. Functional Significance of RNA Markers Associated with the PGR-Negative Serous Ovarian TUMOR Phenotype

The potential targets of piRNAs from Figure 8 and Figure 9 (piR_020813, piR_004307, piR_022437, piR_019914, piR_009295) were predicted as described in our recent manuscript [40]. The list of RNA targets for these piRNAs, represented as RefSeq mRNA accessions, is presented in Table S4 (Sheet 1), which was then converted to gene symbols using the bioDBnet database (https://biodbnet-abcc.ncifcrf.gov/db/db2db.php, last accessed on 15 March 2023). Similarly, potential target mRNAs for hsa-miR-16-5p and hsa-miR-93-5p were identified using the miRtargetlink database (https://ccb-web.cs.uni-saarland.de/mirtargetlink/, last accessed on 15 March 2023). The complete list of miRNA and piRNA gene targets associated with the PGR-negative serous ovarian tumor phenotype is presented in Table S4 (Sheet 2).

**Figure 11:**
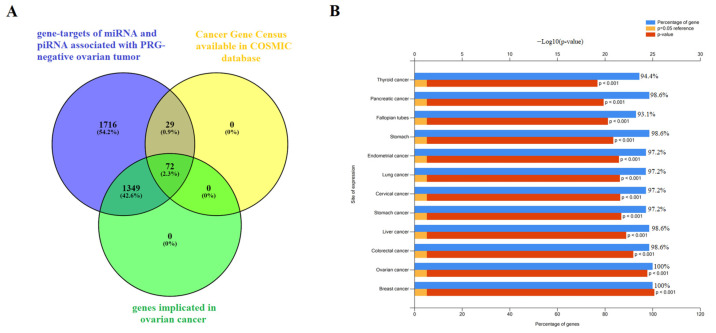
Functional significance of genes potentially regulated by miRNA and piRNA associated with the PGR-negative serous ovarian carcinoma phenotype. (**A**) Venn diagram of gene targets for miRNA and piRNA from logistic regression models shown in Figure 8 and Figure 9 (Table S4, Sheet 2), genes implicated in ovarian cancer according to FunRich3.1.3 (Table S4, Sheet 8), and genes containing mutations that have been causally implicated in cancer according to the Cancer Gene Census from COSMIC database (Table S4, Sheet 11). (**B**) Site of expression of 72 genes, common to three gene lists as shown in (**A**), according to FunRich3.1.3 (Table S4, Sheet 12).

To assess the functional significance of the target genes, we conducted functional enrichment analysis using the FunRich 3.1.3 tool (http://www.funrich.org/download (accessed on 1 March 2023)). The analysis provided valuable insights into the functions and pathways associated with these target genes. Among the 3166 gene targets for miRNA and piRNA from logistic regression models shown in Figure 8 and Figure 9 (Table S4, Sheet 2), 1421 genes were found to be implicated in ovarian cancer (Table S4, Sheet 8), and 101 genes were known to be involved in cancer pathogenesis when mutations occur in them, according to the Cancer Gene Census from the COSMIC database (Table S4, Sheet 11). By comparing these three gene lists using the Venny 2.1 tool (https://bioinfogp.cnb.csic.es/tools/venny/ (accessed on 1 March 2023)), we identified a common set of 72 genes (Figure 11A, Table S4, Sheet 12).

Further analysis of the expression sites of these 72 genes using the FunRich 3.1.3 tool revealed that they are experimentally proven to be involved not only in ovarian cancer but also in various other cancers, such as breast, colorectal, liver, stomach, cervical, lung, endometrial, pancreatic, and thyroid cancers (Figure 11B). This suggests that these genes may play crucial roles in the pathogenesis and development of multiple cancer types, highlighting their potential as significant targets for further investigation and potential therapeutic interventions.

## 3. Discussion

Ovarian cancer is the most lethal gynecologic cancer, with a 5-year survival rate of less than 50 percent [2]. The tumor’s biological properties, including increased invasiveness, chromosomal instability in cancer cells, and chemoresistance, are influenced by the tumor microenvironment, particularly cancer-associated fibroblasts, natural killer (NK) cells, and Th2 cells [14,35,38,39]. The response rate to the immunotherapy already developed and the survival outcomes in serous ovarian carcinomas depend on the immune cell composition in the tumor-associated microenvironment [41]. The low immune reactivity of the tumor is caused by a shift in the Th1/Th2 balance, favoring the prevalence of Th2 cells over Th1 cells [42]. While Th1 cells provide anti-tumor immunity, Th2 cells induce pro-tumorigenic immunity by increasing the presence of immunosuppressive M2 macrophages and promoting the formation of a leaky vasculature, allowing for the unimpeded spread of tumor cells to surrounding tissues [43]. Hormone receptor status has been found to define tumor invasive properties [15,18,44,45,46]. High expression levels of estrogen and progesterone receptors are associated with better outcomes for ovarian cancer patients compared to reduced hormone receptor levels. Conversely, low levels of *PGR* expression are characteristic of aggressive forms of the disease and are linked to poorer outcomes. Additionally, certain polymorphisms in the hormone-binding domain of the *PGR* gene have been associated with an increased risk of ovarian cancer [47,48,49]. Progesterone’s protective effect may be attributed to PGR’s suppression of progesterone receptor membrane component-1 (PGRMC1), which increases the sensitivity of ovarian cancer cells to platinum-based chemotherapy [19]. Therefore, targeted depletion of PGRMC1 could potentially be used as an additional therapy to cisplatin. Recent studies have demonstrated the chemopreventive effect of the synthetic progestin Norethindrone in epithelial ovarian cancer cells (SKOV3). This effect is achieved through the upregulation of *TP53* expression and the downregulation of *VEGF, HIF-1α, COX-2*, and *PGRMC1* expression, leading to significantly reduced SKOV3 cell growth, increased apoptosis and necrosis, and inhibition of cell migration [50].

The aim of the present study was to explore and evaluate candidate factors—specifically, miRNA, piRNA, and mRNA—associated with PGR-negative serous ovarian tumors. The goal was to develop a liquid biopsy test for identifying this aggressive and chemoresistant tumor phenotype before any treatment, and to be prepared to use targeted therapies in addition to conventional treatment regimens.

Significant inverse correlations were found between *PGR* expression levels in ovarian tumor tissues and CA125 serum levels. These CA125 serum levels were significantly inversely correlated with the expression levels of potential regulators miR-199a-5p, miR-214-3p, miR-424-3p, miR-424-5p, and miR-125b-5p, whose target genes include *MUC16, MMP7*, and *MMP9*, as identified through the miRWalk database. Downregulation of miR-199a-5p expression has been associated with ovarian cancer progression, as it showed a negative correlation with tumor infiltration, tumor size, lymphatic metastasis, and TNM stage in ovarian cancer [51]. Additionally, miR-199a-5p downregulation was found in ascites-derived spheroids from the primary tumor site, contributing to the formation of new metastatic niches due to their high invasive capability [52]. Mir-214-3p has a suppressive effect on CDK6 [53] and on MAPK1 [54], and its downregulation promotes cell-cycle progression, proliferation, migration, and invasion of ovarian cancer cells. MiR-424-3p has been shown to sensitize ovarian cancer cells to cisplatin by decreasing the expression of the anti-apoptotic protein galectin-3 [55]. In cases of galectin-3 overexpression, chemoresistance occurs. In ovary cancer cells, the miR-424/503 cluster is silenced by DNA hypermethylation, thereby canceling suppression of the expression of kinesin family member 23 by miR-424-5p and promoting cell proliferation and migration [56]. It is important to note that kinesins play an important regulatory role in the formation of spindles, separation of chromosomes, and cytokinesis, and in the case of abnormal expression/function of kinesins, daughter cells become aneuploidic, thereby resulting in tumorigenesis [57]. In particular, elevated levels of KIF23 have been associated with adverse outcomes in ovarian, breast, and lung cancers [58,59,60]. MiR-125b-5p may serve as a platinum-chemoresistance marker, as its downregulation was observed in tumorspheres derived from the SKOV-3 platinum-resistant cell line [61]. This correlation between chemoresistance and miR-125b-5p expression level may be explained by its direct targeting of BCL2 mRNA, thereby increasing the sensitivity of cancer cells to cisplatin treatment. A similar pattern was demonstrated in gallbladder cancer, where low miR-125b-5p expression and high Bcl2 expression were correlated with poor prognosis [62].

In our study, we observed a significant direct correlation between *MMP7* mRNA levels and the expression levels of *MUC16* mRNA and *MMP9* mRNA. In turn, these levels were inversely correlated with the level of PGR. The downregulation of miR-199a-5p, miR-214-3p, miR-424-3p, miR-424-5p, and miR-125b-5p in PGR-negative serous tumors can lead to increased levels of MUC16, MMP7, and MMP9, thereby enhancing the migratory ability of ovarian cancer cells, facilitating their adhesion to secondary sites, and promoting metastasis [30]. The elevated concentration of MMP7 may be attributed to the induction of *MMP-7* expression through a p38 mitogen-activated protein kinase (MAPK)-dependent pathway, resulting from the interaction between the carboxy-terminal portion of the MUC16/CA125 protein and mesothelin present on mesothelial cells lining the peritoneum [31,32]. Notably, high mesothelin levels have been associated with chemoresistance and poor survival in epithelial ovarian carcinoma, leading to ongoing clinical trials evaluating the safety and efficacy of mesothelin-targeted drugs in platinum-resistant ovarian cancer [63,64,65].

Furthermore, in our study, we found that miR-16-5p, miR-17-5p, miR-20a-5p, and miR-93-5p, responsible for the epithelial–mesenchymal transition (EMT) of the cell [66,67,68,69,70], were significantly directly correlated with blood serum CA125 concentration and significantly inversely correlated with PGR expression levels in tumor tissue. These elevated levels of miRNAs in tumor tissues and peripheral blood of patients with HGSOC serous ovarian cancer are consistent with literature data showing similar changes in biological samples from patients with serous ovarian tumors [16,71,72,73,74]. Notably, a sharp significant decrease in *PGR* mRNA levels was observed in our study in both the LGSOC and HGSOC groups, with a more pronounced drop in the latter. This observation, coupled with the significant increase in expression levels of miR-16-5p, miR-17-5p, miR-20a-5p, and miR-93-5p in the HGSOC group, likely indicates the most aggressive carcinoma phenotype among other types of serous tumors.

We also discovered that the levels of EMT-associated miRNAs significantly directly correlated with the content of hsa_piR_022437, hsa_piR_009295, hsa_piR_020813, hsa_piR_004307, and hsa_piR_019914 in serous ovarian tumor tissues. Among them, the expression level of hsa_piR_004307 showed a significant inverse correlation with PGR expression levels in the tumor. PiRNAs play a role in regulating cell genome stability by interacting in the nucleus with retrotransposon transcripts, forming the RISC complex, which interacts with histone deacetylase, methyltransferase, and DNA methyltransferase, ultimately blocking further retrotransposon transcription to prevent its activity and integration into different parts of the genome [22]. Additionally, piRNAs have known regulatory effects on various signaling pathways in cells, both by destabilizing the target mRNA and inhibiting translation or stabilizing of the target mRNA and activating translation [22,34,75,76]. Analyzing potential target genes of hsa_piR_022437, hsa_piR_009295, hsa_piR_020813, hsa_piR_004307, hsa_piR_019914, and two miRNAs, miR-16-5p and miR-93-5p—which are involved in identifying PGR-negative serous ovarian tumors using logistic regression—revealed the participation of 72 genes in the pathogenesis of ovarian, breast, colorectal, liver, stomach, cervical, lung, endometrial, pancreatic, and thyroid cancers. Some of these genes are associated with mutations, according to the Cancer Gene Census. These findings suggest the presence of common pathogenetic mechanisms in epithelial cancers involving alterations in the functional activity of this group of genes, possibly under the influence of miRNAs and piRNAs, which are used as diagnostic/prognostic parameters in our developed logistic regression models.

The genome sequences coding for certain piRNA classes are often found within protein-coding genes, and functional piRNAs are predominantly produced from the 3′-untranslated regions (3′-UTRs) of mRNA during translation [77]. Furthermore, these mRNA 3′-UTRs, which produce piRNAs, often contain transposon sequences whose activity is regulated by these piRNAs at the post-transcriptional level [78]. Notably, some piRNAs associated with the PGR-negative tumor phenotype are located within the loci of protein-coding genes and/or transposons. For instance, the hsa_piR_022437 DNA sequence is linked to the retrotransposon SINE and located within the *SUN1* gene, which plays a role in directed cell migration [79]. Additionally, the hsa_piR_009295 DNA sequence is located within the centrosome linker protein rootlein (encoded by the *CROCC* gene, also known as *TAX1BP2*), whose overexpression inhibits centrosome duplication, while its depletion leads to centrosome hyperamplification associated with oncogenesis [80,81,82]. Centrosome aberrations may induce the dissemination of metastatic cells and contribute to aggressive cancer subtypes [83].

Regarding the tumor response to adjuvant chemotherapy, we observed that PGR-negative LGSOC and HGSOC showed disease stabilization or progression in 75% and 100% of cases, respectively. This indicates that a significant portion of patients with primary PGR-negative LGSOC and HGSOC are resistant to platinum-based treatment, necessitating novel therapeutic and preventive approaches. In our study, we developed a liquid biopsy method based on regression analysis of hsa_piR_022437 and hsa-miR-16-5p content in the blood plasma of patients. This method can be utilized for diagnosing the PGR-negative tumor phenotype with 86% sensitivity before surgery and chemotherapy, thereby enabling the selection of the appropriate treatment strategy for this highly aggressive type of ovarian cancer. Moreover, we devised a model based on the quantitation of hsa_piR_020813 and hsa-miR-17-5p in blood plasma to prognosticate chemoresistance in LGSOC and HGSOC with 85.71% sensitivity. The role of miR-17-5p upregulation in chemoresistance and oncogenesis has been previously demonstrated, as it targets E3 ubiquitin-protein ligase *TRIM8* and the anti-apoptotic p21 protein *CDKN1A* genes [84,85]. Recent research involved conducting a trial to investigate the effect of adding hyperthermic intraperitoneal chemotherapy (HIPEC) to interval cytoreductive surgery in patients with stage III epithelial ovarian cancer who were receiving neoadjuvant chemotherapy. This procedure resulted in longer recurrence-free survival and overall survival compared to surgery alone [86]. It is possible that—in addition to administering HIPEC with cisplatin—adding targeted therapy aimed at depleting PGRMC1, reducing mesothelin expression, and inhibiting Th2 infiltration could enhance the effectiveness of treatment and improve the longevity of patients with highly aggressive serous ovarian tumors of the PGR-negative type.

**Figure 12:**
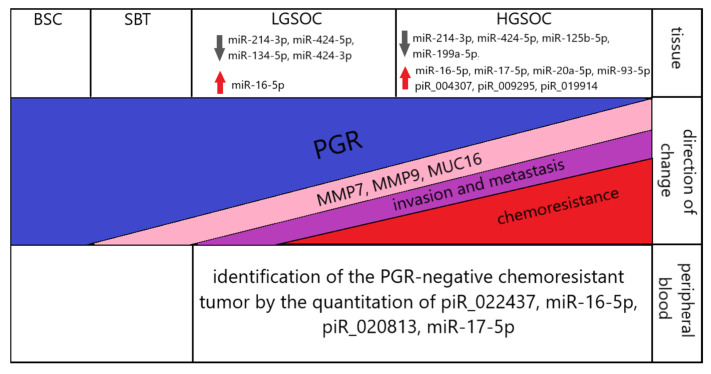
Summary of the data obtained. Up and down arrows indicate the corresponding significant increase and decrease in the level of expression of small non-coding RNAs in serous ovarian carcinomas (LGSOC and HGSOC) relative to BSC.

A scheme summarizing the molecular biological properties of the analyzed serous ovarian tumors is presented in Figure 12.

## 4. Materials and Methods

### 4.1. Patients Enrolled in the Study

A total of 38 women enrolled in the study, aged between 29 and 71 years old with primary ovarian tumors, were referred to the National Medical Research Center for Obstetrics, Gynecology, and Perinatology, named after Academician V.I. Kulakov of the Ministry of Healthcare of the Russian Federation, for clinical and instrumental additional examination and surgical intervention in the volume depending on the stage of the disease, which was histologically verified according to FIGO. After adjuvant chemotherapy (carboplatin AUC 6 + paclitaxel 175 mg/m^2^), tumor response was evaluated according to the RECIST 1.1 criteria. The following groups were formed: benign serous cystadenoma, n = 10; borderline serous cystadenoma, n = 6; low-grade serous ovary cancer, n = 8; high-grade serous ovary cancer, n = 14.

### 4.2. RNA Isolation from Peripheral Blood Plasma

S-MONOVETTE tubes containing EDTA KE (Sarstedt AG & Co., Ltd., Nümbrecht, Germany, cat. No. 04.1915.100) were used to sample venous blood from patients entrolled in the study. A 200 µL volume of blood plasma, collected after two-step centrifugation for 20 min at 300× *g* (4 °C) and for 10 min at 16,000× *g*, was used for RNA extraction applying an miRNeasy Serum/Plasma Kit (Qiagen, Germany, cat. No. 217184).

### 4.3. RNA Isolation from Ovarian Tumors

Ovarian tumor samples were collected during surgery and immediately frozen in liquid nitrogen or embedded in paraffin blocks after fixation with neutral formalin; in the former case, for subsequent total RNA extraction using the miRNeasy Micro Kit (Qiagen, Hilden, Germany, catalog No. 217084) followed by the RNeasy MinElute Cleanup Kit (Qiagen, Germany, catalog No. 74204), or in the latter case using deparaffinization solution (Qiagen, Hilden, Germany, catalog No. 19093) and the RNeasy FFPE Kit (Qiagen, Hilden, Germany, catalog No. 73504). A Qubit fluorometer 3.0 (Life Technologies, Petaling Jaya, Malaysia, cat. Q33216) was used for RNA concentration measurement. Total RNA quality was examined on the Agilent Bioanalyzer 2100 (Agilent, Waldbronn, Germany, cat. No. G2939A) using the RNA 6000 Nano Kit (Agilent Technologies, Santa Clara, CA, USA, cat. No. 5067-1511).

### 4.4. Small RNA Deep Sequencing

A 500-ng amount of total RNA from frozen tumor tissues was used for cDNA libraries synthesis applying the NEBNext^®^ Multiplex Small RNA Library Prep Set for Illumina^®^ (Set11 and Set2, New England Biolab^®^, Frankfurt am Main, Germany, cat. No. E7300S, E7580S). After amplification for 14 PCR cycles and purification in the 6% polyacrylamide gel, cDNA libraries were sequenced on the NextSeq 500 platform (Illumina, San Diego, AC, USA, cat. No. SY-415-1001). Deep sequencing data were processed as described in our previous publication [40], using Cutadapt to remove adapters; bowtie aligner [87] to map all trimmed reads in the range of 16–50 bp to the GRCh38.p15 human genomes, miRBase v21, and piRNABase; featureCount tool from the Subread package [88] to count aligned reads; and the DESeq2 package [89] to carry out differential expression analysis of the sncRNA.

### 4.5. Reverse Transcription and Quantitative Real-Time PCR of Small Noncoding RNA

Seven microliters of total RNA obtained in Section 4.2 or 250 ng of total RNA from FFPE samples obtained in Section 4.3 were converted into cDNA in accordance with the miScript^®^ II RT Kit protocol (Qiagen, Germany, cat. No. 218161). After completion of the reaction and dilution of the sample by 20 times, cDNA (2 µL) was amplified during real-time PCR using a forward primer specific to the studied RNA (Table S5) and the miScript SYBR Green PCR Kit (Qiagen, Germany, cat. No. 218075). The following PCR conditions for miRNA and piRNA amplification were used: (1) 15 min at 95 °C and (2) 40 cycles at 94 °C for 15 s, an optimized annealing temperature (46.2–62 °C) for 30 s, and 70 °C for 30 s in a StepOnePlusTM thermocycler (Applied Biosystems, Waltham, MA, USA, cat. No. 4376600). miR-30d-5p was used as the reference RNA to quantify miRNA and hsa_piR_004308 was used as the reference RNA to quantify piRNA in the blood plasma sample by the ∆Ct method. The relative expression of miRNA and piRNA in the FFPE ovarian tumor sections was determined by the ∆Ct method using SNORD68 as the reference RNA.

### 4.6. Reverse Transcription and Quantitative Real-Time PCR of mRNA

A 125 ng amount of total RNA from FFPE ovarian tumor sections was converted into cDNA in a reaction mixture (25 µL) containing 10 µM random hexameric primer (Evrogen, Moscow, Russia), 1× M-MLV RT buffer (M531A, Promega, Madison, WI, USA), 1× dNTP mix (0.2 mM each, Evrogen, Moscow, Russia), and 200 U M-MLV reverse transcriptase (M1708, Promega, Madison, WI, USA) at 37 °C over 60 min, followed by incubation at 95 °C over 10 min; then, the sample volume was adjusted with deionized water to 100 µL. The synthesized cDNA (2 µL) was used as a template for real-time PCR in a reaction mixture (20 µL) containing 150 nM each of the forward and reverse primers specific to the studied mRNA (Table S5) in a 1× qPCRmix-HS SYBR + HighROX (Evrogen, Moscow, Russia). The following PCR conditions were used: (1) 5 min at 95 °C and (2) 40 cycles at 95 °C for 20 s, an optimized annealing temperature (48.9–63.8 °C) for 20 s, and 72 °C for 30 s in a CFX96 Real-Time System (C1000 Touch Thermal Cycler plus CFX96 Optics Module, BioRad, Singapore). The relative expression of mRNA was determined by the ∆Ct method using the geometric mean of *ACTB*, *TUBA*, and *GAPDH* as the reference RNAs. 

### 4.7. Immunohistochemistry

PGR immunohistochemical staining of formalin-fixed paraffin-embedded specimens of serous ovarian tumors was performed as described in our previous publication [16] according to the Allred scale [90]. Monoclonal antibodies against PgR (clone 1E2) manufactured by Ventana were used, recognized as A and B isoforms of PgR. An automated immunostainer, the Ventana Benchmark Ultra, and the prescribed Ventana protocol for progesterone receptor (PgR) staining were used. Appropriate controls were included.

### 4.8. Statistical Analysis of the Obtained Data

Scripts written in R language [88] and RStudio [91] were used for statistical processing as described in our previous publication [16] applying the Shapiro–Wilk test, the Mann–Whitney test for paired comparison, Spearman’s nonparametric correlation test, and logistic regression analysis. Study results were considered reliable if the value of statistical significance (*p*) was less than 0.05.

## Figures and Tables

**Table 1:** Sample characteristics of the patients with serous ovary tumors.

Patient ID	Age, Years	FIGO	1—Primary Tumor Resection, 2—Complete Cytoreduction (Size of Residual Tumor Foci Less Than 2.5 mm), 3—Suboptimal Cytoreduction (Size of Residual Tumor Foci 2.5 mm–2.5 cm)	RECIST 1.1 MRI/CT Criteria: 1—Complete Response, 2—Partial Response, 3—Stable Disease, 4—Progressive Disease	Diagnosis	ID, FFPE Sample	Progesterone Receptor Expression in Tumor, Allred Score *	ID, Blood Plasma Sample	CA 125 Level before Treatment, U/ml
P1	34	-	1	-	BSC	1	8	959	12
P2	41	-	1	-	BSC	7	8	724	18
P3	45	-	1	-	BSC	10	8	957	26
P4	53	-	1	-	BSC	17	8	802	12
P5	36	-	1	-	BSC	18	8	806	3
P6	46	-	1	-	BSC	24	8	866	3
P7	48	-	1	-	BSC	26	8	849	19
P8	43	-	1	-	BSC	31	8	908	11
P9	38	-	1	-	BSC	32	8	745	4
P10	45	-	1	-	BSC	37	8	705	9
P11	32	IA	1	-	SBT	2	7	956	64
P12	35	IA	1	-	SBT	22	8	453	15
P13	43	IA	1	-	SBT	25	7	900	9
P14	36	IA	1	-	SBT	27	7	510	55
P15	39	IB	1	-	SBT	33	7	817	12
P16	43	IA	1	-	SBT	38	6	685	3
P17	34	IIIC	2	1	LGSOC	16	4	686	45
P18	54	IIIC	3	4	LGSOC	34	0	752	521
P19	46	IIIC	3	3	LGSOC	5	0	1004	41
P20	30	IIIC	3	3	LGSOC	6	0	554	604
P21	45	IIIC	2	1	LGSOC	8	4	731	173
P22	29	IIIC	2	4	LGSOC	15	5	796	550
P23	40	IIB	2	1	LGSOC	28	6	729	441
P24	53	IIIA	2	1	LGSOC	29	6	965	372
P25	63	IIIC	3	4	HGSOC	3	0	19	42
P26	51	IIIC	2	4	HGSOC	4	0	448	3808
P27	38	IIIC	3	4	HGSOC	9	0	2008	1244
P28	71	IIIC	3	4	HGSOC	11	0	13	517
P29	33	IIIC	3	2	HGSOC	12	6	939	59
P30	51	IIIC	2	3	HGSOC	13	0	679	2000
P31	45	IIB	2	3	HGSOC	14	0	782	517
P32	48	IC	2	4	HGSOC	19	0	11	190
P33	41	IIIC	2	1	HGSOC	20	3	672	1088
P34	54	IIIC	3	4	HGSOC	21	0	649	200
P35	42	IIIC	2	1	HGSOC	23	3	684	1293
P36	57	IIC	3	4	HGSOC	30	0	15	198
P37	77	IIIC	2	2	HGSOC	35	4	1060	1203
P38	45	IIA	2	3	HGSOC	36	0	22	60

* The Allred score combines the percentage of positive cells and the intensity of the reaction product in most of the carcinoma. Scores of 0–2 are considered negative. Scores of 3–8 are considered positive.

**Table 2:** Comparison of SBT, LGSOC, and HGSOC groups relative to BSC by miRNA expression levels in serous ovarian tumors.

miRNA	Group	Me, −∆Ct	Q1	Q3	Wilcoxon–Mann–Whitney Test, *p*-Value	Potential Gene-Target
miR-125b-5p	BSC	1.79	0.83	2.91		*MUC16*, *MMP7*
	SBT	1.46	0.6	2.18	0.492258	
	LGSOC	0.79	0.68	0.89	0.101102	
	HGSOC	0.57	−0.49	1.45	0.022015	
miR-134-5p	BSC	−8.35	−8.95	−7.64		*MUC16*, *MMP9*
	SBT	−8.78	−9.1	−8.38	0.367632	
	LGSOC	−9.9	−10.45	−9.17	0.026647	
	HGSOC	−9.06	−9.71	−8.27	0.234983	
miR-139-5p	BSC	−8.19	−10.03	−7.12		*MMP9*
	SBT	−8.81	−10.7	−8.57	0.635365	
	LGSOC	−8.15	−8.94	−7.29	0.572604	
	HGSOC	−8.5	−9.3	−7.77	0.752095	
miR-199a-5p	BSC	−4.5	−5.05	−3.72		*MUC16*, *MMP7*
	SBT	−4.96	−5.58	−4.16	0.562188	
	LGSOC	−5.83	−6.35	−4.51	0.067599	
	HGSOC	−5.73	−6.41	−5.17	0.007251	
miR-214-3p	BSC	−2.43	−3.24	−1.93		*MUC16*, *MMP7*
	SBT	−3.1	−4.08	−2.29	0.263487	
	LGSOC	−4.55	−4.73	−3.38	0.006216	
	HGSOC	−4.09	−4.84	−3.67	0.000274	
miR-424-3p	BSC	−6.63	−7.45	−5.93		*MUC16*, *MMP9*
	SBT	−7.34	−8.54	−6.37	0.313187	
	LGSOC	−8.66	−9.23	−8.19	0.006216	
	HGSOC	−7.25	−8.67	−6.75	0.137503	
miR-424-5p	BSC	−2.91	−3.63	−1.89		*MUC16*
	SBT	−3.57	−4.74	−2.23	0.492258	
	LGSOC	−4.94	−5.39	−4.65	0.001371	
	HGSOC	−3.86	−4.65	−3.52	0.041717	

**Table 3:** Comparison of SBT, LGSOC, and HGSOC relative to BSC by mRNA expression level in serous ovarian tumors.

mRNA	Group	Me, −∆Ct	Q1	Q3	Wilcoxon–Mann–Whitney Test, *p*-Value
*MMP7*	BSC	−6.19	−7.14	−4.09	
	SBT	−1.84	−2.05	−1.45	0.041958
	LGSOC	−1.79	−3.78	−1.07	0.034279
	HGSOC	−2.59	−2.89	−1.45	0.030588
*MMP9*	BSC	−6.86	−7.24	−6.05	
	SBT	−6.74	−7.88	−5.23	0.874875
	LGSOC	−5.29	−5.89	−4.72	0.011655
	HGSOC	−4.18	−5.22	−2.16	0.022015
*MUC16*	BSC	−3.96	−5.3	−2.37	
	SBT	0.32	−0.61	0.68	0.031219
	LGSOC	0.03	−0.57	0.75	0.002057
	HGSOC	−1.86	−3.23	−0.94	0.018545
*PGR*	BSC	0.78	0.5	1.5	
	SBT	−0.23	−0.84	0.47	0.093407
	LGSOC	−2.83	−4.43	−1.95	0.000183
	HGSOC	−4.02	−4.97	−3.03	3.06 × 10^−5^

**Table 4:** Comparative analysis of the expression level of miRNAs implicated in EMT in the SBT, LGSOC, and HGSOC relative to BSC groups.

miRNA	Group	Me, −∆Ct	Q1	Q3	Wilcoxon–Mann–Whitney Test, *p*-Value
miR-16-5p	BSC	−4.76	−5.75	−3.94	
	SBT	−4.07	−4.65	−3.44	0.263487
	LGSOC	−3.32	−4.03	−3.07	0.034279
	HGSOC	−2.21	−3.32	−1.46	9.89 × 10^−5^
miR-17-5p	BSC	−5.9	−6.86	−5.42	
	SBT	−5.04	−5.42	−4.49	0.072677
	LGSOC	−5.81	−5.86	−4.54	0.274281
	HGSOC	−2.97	−3.81	−2.45	3.06 × 10^−5^
miR-20a-5p	BSC	−5.57	−6.51	−5.17	
	SBT	−4.79	−5.58	−4.34	0.313187
	LGSOC	−5.03	−5.72	−4.18	0.236985
	HGSOC	−2.86	−3.69	−2.36	0.000504
miR-93-5p	BSC	−8.37	−8.97	−7.23	
	SBT	−6.89	−7.51	−6.67	0.093407
	LGSOC	−7	−7.31	−6.38	0.067599
	HGSOC	−4.81	−5.4	−4.15	3.06 × 10^−5^

**Table 5:** Comparative analysis of the piRNA expression level in the SBT, LGSOC, and HGSOC relative to BSC groups in FFPE sections of tumor tissues.

piRNA	Group	Me, −∆Ct	Q1	Q3	Wilcoxon–Mann–Whitney Test, *p*-Value
hsa_piR_004307	BSC	−1.92	−2.92	−1.08	
	SBT	−1.03	−1.58	−0.75	0.21978
	LGSOC	−1.7	−2.49	−1.12	0.761826
	HGSOC	−0.14	−0.8	0.27	0.003067
hsa_piR_009295	BSC	0.09	−0.97	1.83	
	SBT	0.76	0.47	1.67	0.492258
	LGSOC	−0.33	−1.2	1.66	0.696467
	HGSOC	1.92	0.84	2.67	0.022015
hsa_piR_019914	BSC	−1.43	−2.41	0.27	
	SBT	−0.44	−0.57	0.12	0.427822
	LGSOC	−1.89	−2.28	−1.07	0.828557
	HGSOC	0.64	−0.57	1.04	0.015536
hsa_piR_020813	BSC	−0.82	−1.07	−0.42	
	SBT	−0.48	−0.6	−0.4	0.562188
	LGSOC	−1.06	−1.24	−0.66	0.359934
	HGSOC	0.41	−0.94	1.21	0.137503
hsa_piR_022437	BSC	−1.81	−2.42	−1.14	
	SBT	−1.73	−2.18	−1.51	1
	LGSOC	−3.2	−3.34	−2.44	0.083139
	HGSOC	−0.87	−2.14	−0.2	0.154081

**Table 6:** Parameters of logistic regression models in Figure 8.

Coefficient	Coefficient Value (95% CI)	Wald Test	*p*-Value	Odds Ratio (95% CI)	Sensitivity	Specificity
model 1					0.7857	0.9167
(Intercept)	6.549 (2.224; 12.346)	2.618	0.008	698.816 (9.245; 230,163.148)		
piR_020813	−1.504 (−3.151; −0.316)	−2.144	0.032	0.222 (0.042; 0.728)		
piR_004307	1.142 (0.198; 2.475)	2.045	0.04	3.136 (1.219; 11.885)		
miR-93-5p	1.037 (0.34; 1.935)	2.614	0.008	2.823 (1.405; 6.928)		
model 2					0.9286	0.7083
(Intercept)	6.701 (2.428; 13.488)	2.429	0.015	813.662 (11.341; 720,717.037)		
piR_020813	−1.807 (−3.818; −0.415)	−2.127	0.033	0.164 (0.021; 0.66)		
miR-16-5p	1.803 (0.678; 3.517)	2.56	0.01	6.073 (1.97; 33.695)		
piR_022437	1.248 (0.259; 2.713)	2.063	0.039	3.483 (1.296; 15.081)		
model 3					0.9286	0.75
(Intercept)	6.803 (2.115; 13.645)	2.397	0.016	900.756 (8.292; 844,027.632)		
piR_004307	2.748 (0.756; 5.757)	2.23	0.025	15.613 (2.13; 316.434)		
piR_019914	−2.168 (−4.702; −0.446)	−2.046	0.04	0.114 (0.009; 0.639)		
miR-93-5p	0.881 (0.209; 1.811)	2.248	0.024	2.415 (1.233; 6.12)		
model 4					0.8571	0.875
(Intercept)	4.319 (1.219; 8.825)	2.29	0.021	75.133 (3.384; 6806.095)		
piR_020813	−1.541 (−3.308; −0.29)	−2.051	0.04	0.214 (0.036; 0.747)		
piR_004307	1.129 (0.129; 2.466)	1.951	0.051	3.093 (1.138; 11.782)		
miR-16-5p	1.327 (0.374; 2.65)	2.364	0.018	3.773 (1.454; 14.161)		
model 5					0.8571	0.75
(Intercept)	7.585 (2.542; 14.208)	2.603	0.009	1969.531 (12.709; 1,481,859.036)		
piR_009295	−1.37 (−2.869; −0.194)	−2.061	0.039	0.254 (0.056; 0.823)		
piR_004307	1.901 (0.452; 3.84)	2.276	0.022	6.698 (1.572; 46.542)		
miR-93-5p	0.772 (0.174; 1.53)	2.3	0.021	2.164 (1.191; 4.619)		
model 6					0.7857	0.875
(Intercept)	6.903 (2.61; 12.621)	2.769	0.005	995.67 (13.604; 302,894.002)		
piR_020813	−1.077 (−2.305; −0.093)	−1.965	0.049	0.34 (0.099; 0.91)		
miR-93-5p	1.071 (0.382; 1.977)	2.697	0.006	2.92 (1.466; 7.221)		
piR_022437	0.72 (0.006; 1.611)	1.819	0.068	2.054 (1.006; 5.008)		

**Table 7:** Parameters of logistic regression models in Figure 9.

Coefficient	Coefficient Value (95% CI)	Wald Test	*p*-Value	Odds Ratio (95% CI)	Sensitivity	Specificity
model 1					0.8571	0.9583
(Intercept)	−4.296 (−7.698; −1.991)	−3.048	0.002	0.013 (0.0004; 0.136)		
piR_022437	1.288 (0.399; 2.683)	2.312	0.02	3.628 (1.49; 14.64)		
miR-16-5p	0.746 (0.137; 1.531)	2.173	0.029	2.109 (1.147; 4.625)		
model 2					0.7857	0.8333
(Intercept)	−2.084 (−3.604; −0.936)	−3.138	0.001	0.124 (0.027; 0.392)		
piR_022437	1.384 (0.6; 2.561)	2.861	0.004	3.991 (1.822; 12.957)		
model 3					0.7143	0.875
(Intercept)	−3.326 (−5.932; −1.428)	−2.956	0.003	0.035 (0.002; 0.239)		
miR-16-5p	0.921 (0.36; 1.671)	2.815	0.004	2.513 (1.434; 5.319)		

**Table 8:** Parameters of logistic regression models in Figure 10.

Coefficient	Coefficient Value (95% CI)	Wald Test	*p*-Value	Odds Ratio (95% CI)	Sensitivity	Specificity
model 1					0.8571	0.875
(Intercept)	1.074 (−1.537; 4.13)	0.789	0.43	2.926 (0.215; 62.164)		
piR_022437	1.248 (0.132; 3.279)	1.653	0.098	3.484 (1.141; 26.543)		
piR_009295	0.359 (−0.092; 1.013)	1.38	0.167	1.432 (0.912; 2.754)		
model 2					0.8571	0.875
(Intercept)	1.383 (−1.465; 5.397)	0.854	0.393	3.986 (0.231; 220.636)		
piR_022437	1.064 (0.145; 2.606)	1.826	0.068	2.897 (1.157; 13.55)		
miR-17-5p	0.549 (−0.112; 1.536)	1.408	0.159	1.732 (0.894; 4.646)		
model 3					0.8571	0.75
(Intercept)	6.289 (2.007; 14.752)	2.061	0.039	538.475 (7.439; 2,550,730.387)		
piR_020813	0.266 (0.033; 0.66)	1.787	0.074	1.305 (1.034; 1.934)		
miR-17-5p	1.047 (0.252; 2.555)	1.896	0.058	2.85 (1.287; 12.867)		
model 4					0.7143	1
(Intercept)	−1.09 (−2.878; 0.348)	−1.377	0.169	0.336 (0.056; 1.416)		
piR_022437	1.563 (0.479; 3.243)	2.338	0.019	4.771 (1.614; 25.607)		
piR_004307	−0.752 (−2.184; 0.365)	−1.219	0.223	0.471 (0.113; 1.441)		
model 5					0.6429	1
(Intercept)	3.565 (1.113; 7.275)	2.365	0.018	35.345 (3.045; 1444.262)		
piR_009295	0.343 (−0.061; 0.867)	1.516	0.13	1.41 (0.941; 2.38)		
miR-17-5p	0.472 (−0.097; 1.244)	1.469	0.142	1.604 (0.907; 3.471)		
model 6					0.7857	0.875
(Intercept)	0.723 (−2.03; 4.213)	0.487	0.626	2.061 (0.131; 67.544)		
piR_022437	1.032 (0.048; 2.641)	1.667	0.095	2.807 (1.049; 14.027)		
miR-93-5p	0.35 (−0.25; 1.138)	1.056	0.291	1.419 (0.779; 3.12)		
model 7					0.7857	0.875
(Intercept)	−0.049 (−2.18; 2.235)	−0.047	0.962	0.952 (0.113; 9.347)		
piR_022437	1.067 (0.139; 2.613)	1.805	0.071	2.907 (1.149; 13.633)		
miR-20a-5p	0.206 (−0.332; 0.796)	0.76	0.447	1.228 (0.718; 2.217)		
model 8					0.9286	0.75
(Intercept)	3.48 (0.899; 7.513)	2.148	0.032	32.471 (2.457; 1830.906)		
piR_020813	0.116 (−0.069; 0.344)	1.167	0.243	1.123 (0.934; 1.41)		
miR-93-5p	0.588 (0.018; 1.385)	1.774	0.076	1.801 (1.018; 3.996)		
model 9					0.7857	1
(Intercept)	−0.643 (−2.153; 0.614)	−0.947	0.144	0.525 (0.116; 1.847)		
piR_022437	1.199 (0.3; 2.736)	2.048	0.041	3.315 (1.35; 15.418)		

## Data Availability

Not applicable.

## Supplementary Materials

The following supporting information can be downloaded at: https://www.mdpi.com/article/10.3390/ijms241512214/s1.
