# Supplementary material for: Blood Plasma Small Non-Coding RNAs as Diagnostic Molecules for the Progesterone-Receptor-Negative Phenotype of Serous Ovarian Tumors
Source: Int J Mol Sci. 2023 Jul 30;24(15):12214. doi: 10.3390/ijms241512214 (PMC10419267; doi:10.3390/ijms241512214)
Supplement: Supplementary file 1 [file ijms-24-12214-s001.zip › ijms-2529473-supplementary.pdf]

Table S1: miRNA deep sequencing data for serous ovarian tumors.

Table S2: piRNA deep sequencing data for serous ovarian tumors.

Table S3: Spearman's nonparametric correlation test data.

Table S4: functional analysis of gene-targets for piRNAs and miRNAs from Figure 7 and Figure 8.

Table S5: parameters of PCR primers used to analyze miRNA, piRNA and mRNA.
